# Supplementary material for: Characterization of programmed cell death pathways activated in Mycobacterium tuberculosis-infected human macrophages
Source: Cell Death Discov. 2026 Jun 8;12:334. doi: 10.1038/s41420-026-03156-1 (PMC13470034; doi:10.1038/s41420-026-03156-1)
Supplement: Supplementary file 1 [file 41420_2026_3156_MOESM1_ESM.pdf]

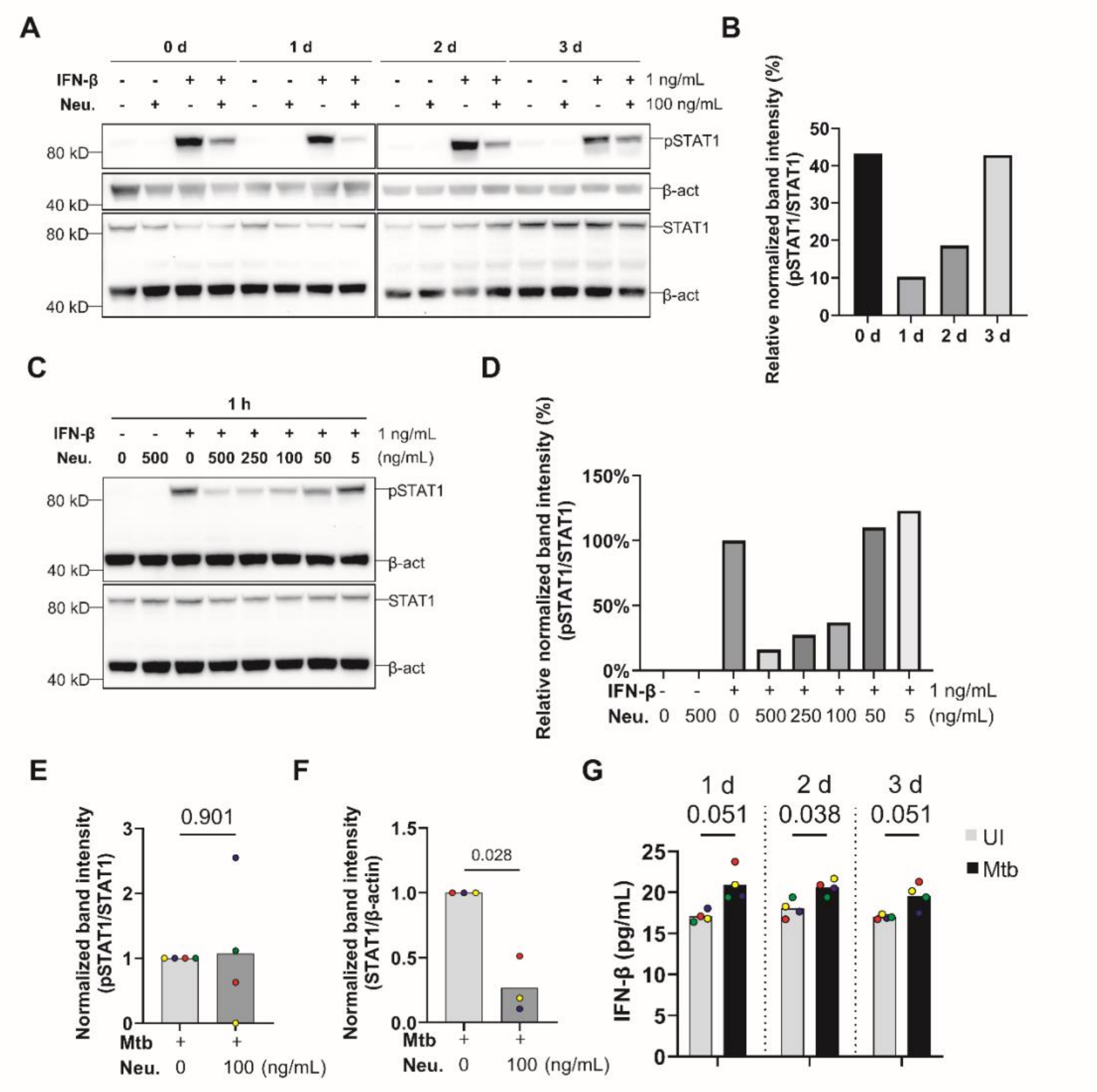

Fig. S1: Optimization of the Interferon-alpha/beta receptor (IFNAR) neutralizing assay. A Immunoblot images showing STAT1 expression and phosphorylation in THP-1 cells following IFN-β treatment and IFNAR neutralization at various time points. Images are from a single experiment (N = 1). **B** Quantification of band intensities shown in **A**. **C** Western blot images showing STAT1 expression and phosphorylation in response to increasing concentrations of IFN-β and IFNAR neutralization. Images are from a single experiment (N = 1). **D** Quantification of band intensities shown in **C**. **E, F** Quantification corresponding to Fig. 1D, showing the relative ratio of p-STAT1α to STAT1 (**E**) and the normalized STAT1/β-actin ratio (**F**). For **F**, Grubb's test identifies and excludes an outlier at α = 0.05. **G** THP-1 cells were infected with H37Rv Mtb at MOI: 5 for 1–3 days. IFN-β secretion was measured by enzyme-linked immunosorbent assay (ELISA). For **E**, **F**, and **G**, each colored dot represents an independent experiment (N = 4). Welch's t-tests were used for statistical analysis, and *p*-values are shown on the graphs.

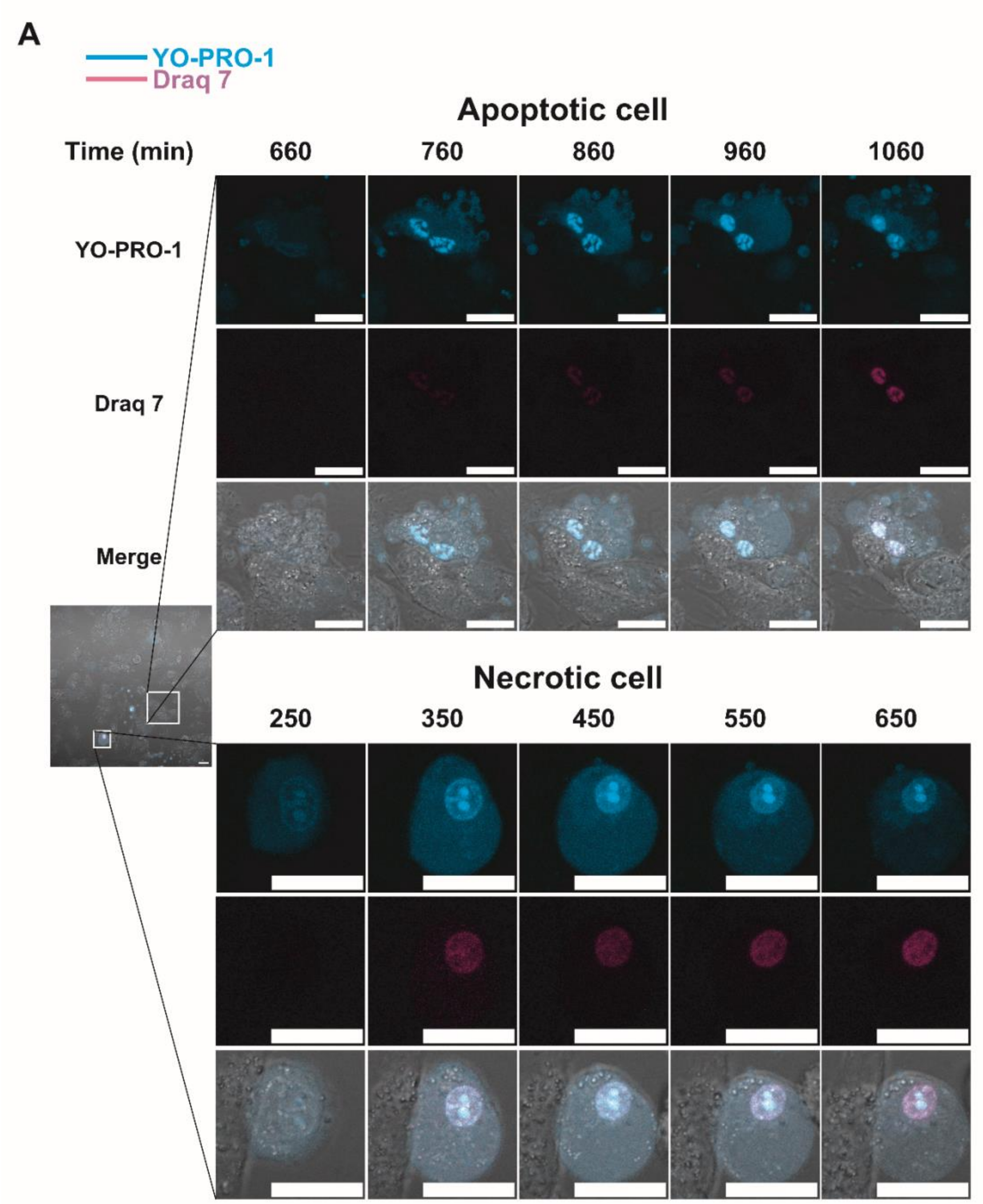

Fig. S2: Validation of YO-PRO-1 and Draq7 staining in distinct cell death modalities. **A** Representative confocal fluorescence images of THP-1 cells treated with 10 μM MG132 (N = 1). Following MG132 addition, cells were stained with YO-PRO-1 (100 nM) and Draq7 (300 nM) for 15 min before initiating time-lapse live-cell imaging. Cells were maintained at 37 °C and 5% CO_2 and imaged continuously for 20 h. Z-stack images (11 slices at 1 μm intervals) were captured every 10 min. Regions containing cells exhibiting characteristic apoptotic or necrotic morphology were selected and enlarged. Fluorescence signals for YO-PRO-1 (cyan), Draq7 (magenta), and merged images with transmitted-light overlays are shown. Five representative time points are displayed for each condition. The necrotic-cell image layout is identical to that of the apoptotic-cell panel. Scale bars: 20 μm.

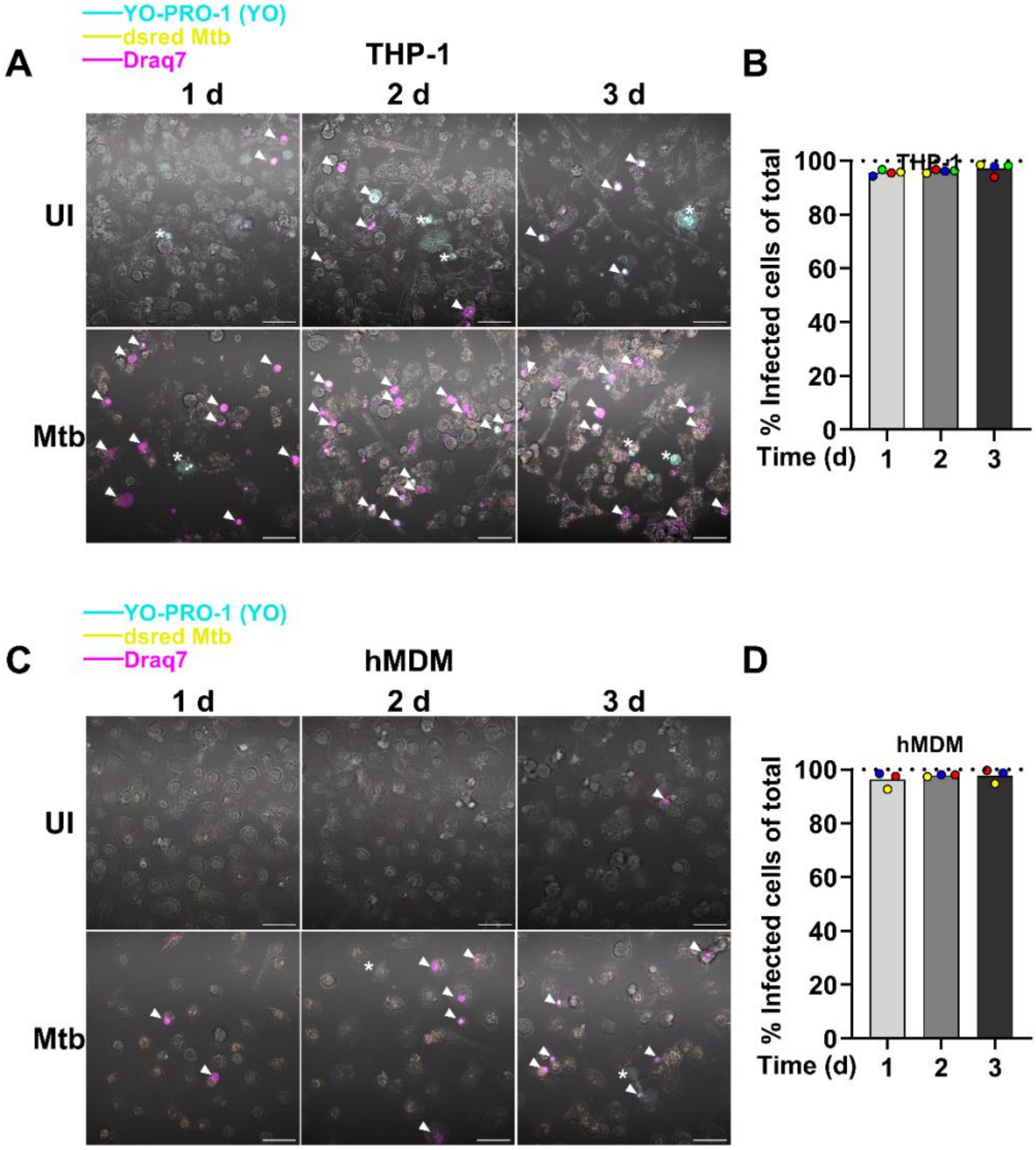

Fig. S3: Assessing early apoptosis by YO-PRO-1 and Draq7 staining in Mtb-infected THP-1 cells. A, C Representative confocal fluorescence images of THP-1 (**A**) or hMDMs (**C**). THP-1 cells (N = 4) or hMDMs (N = 3) were infected with dsRed H37Rv Mtb at MOI:5 for 1, 2, or 3 d. Cells were stained with YO-PRO-1 and Draq 7 for 15 min at 37 °C prior to imaging. Z-stack images (11 slices at 1 µm intervals) were captured for each field of view (FoV). White triangles indicate necrotic cells, while asterisks indicate early apoptotic cells. Fluorescence signals for YO-PRO-1 (cyan), Draq7 (magenta), dsred (yellow), and transmitted-light signals are merged. Scale bars: 50 µm. **B, D** Quantification of percentages of infected cells among total cells for THP-1 cells (**B**) or hMDMs (**D**).

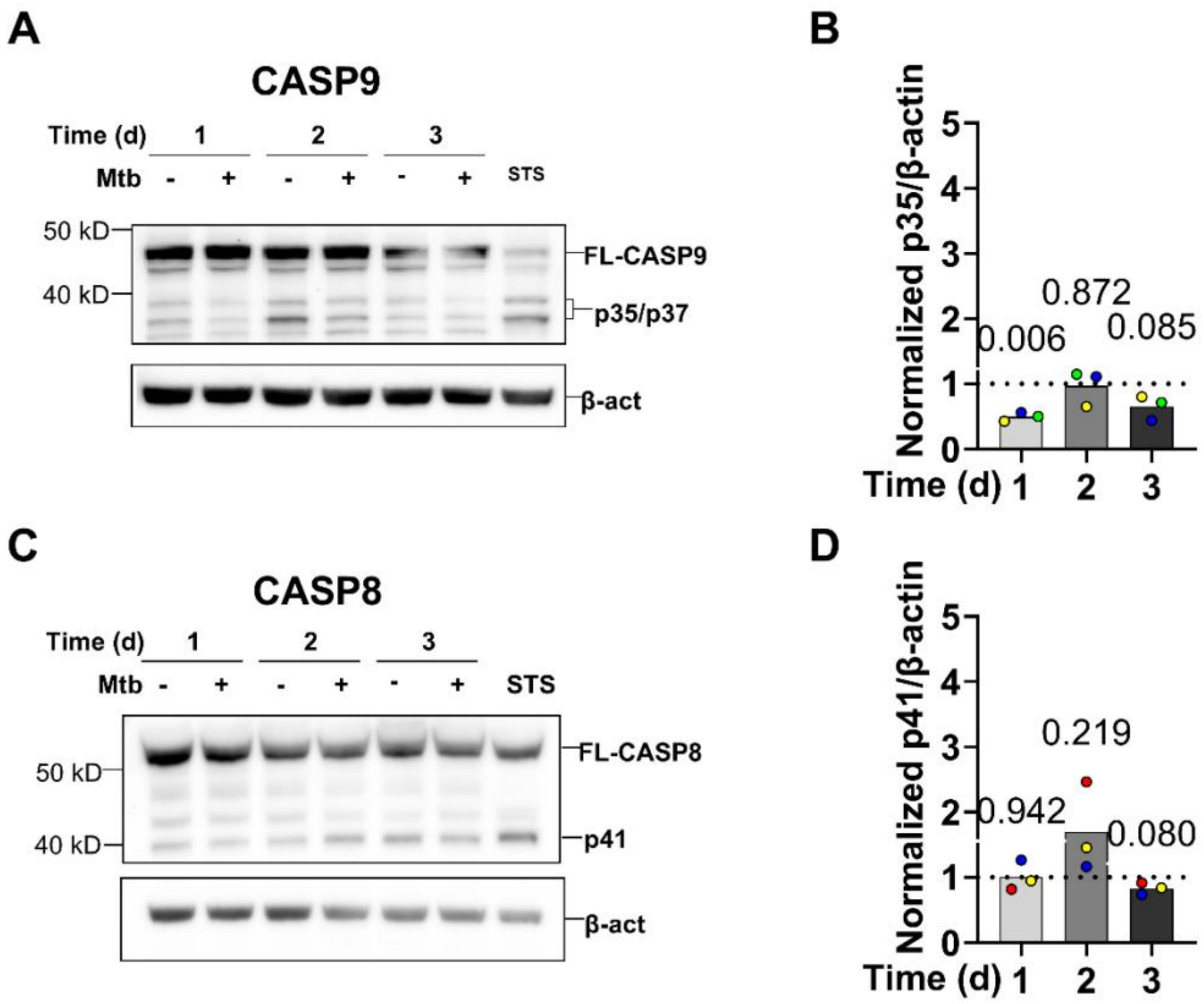

Fig. S4: Mtb H37Rv does not induce CASP8 or 9 activation in THP-1 cells. A, C Representative western blot images showing the cleavage of CASP9 and CASP8, respectively. THP-1 cells were infected with H37Rv Mtb (+) at MOI:5 for 1-3 days or left uninfected (-). STS: Staurosporine (1 μM, 6 h). **B, D** Bar graphs showing the quantification of normalized band intensities corresponding to THP-1 cleaved CASP9 (**B**), and CASP8 (**D**). Each value in the infected groups was normalized to its uninfected counterpart from the same experiment. Band intensities were quantified using ImageLab software. One-sample t-tests were performed to compare the normalized values to 1, and *p*-values are shown in the graphs. Each colored data point represents an independent experiment (N = 3).

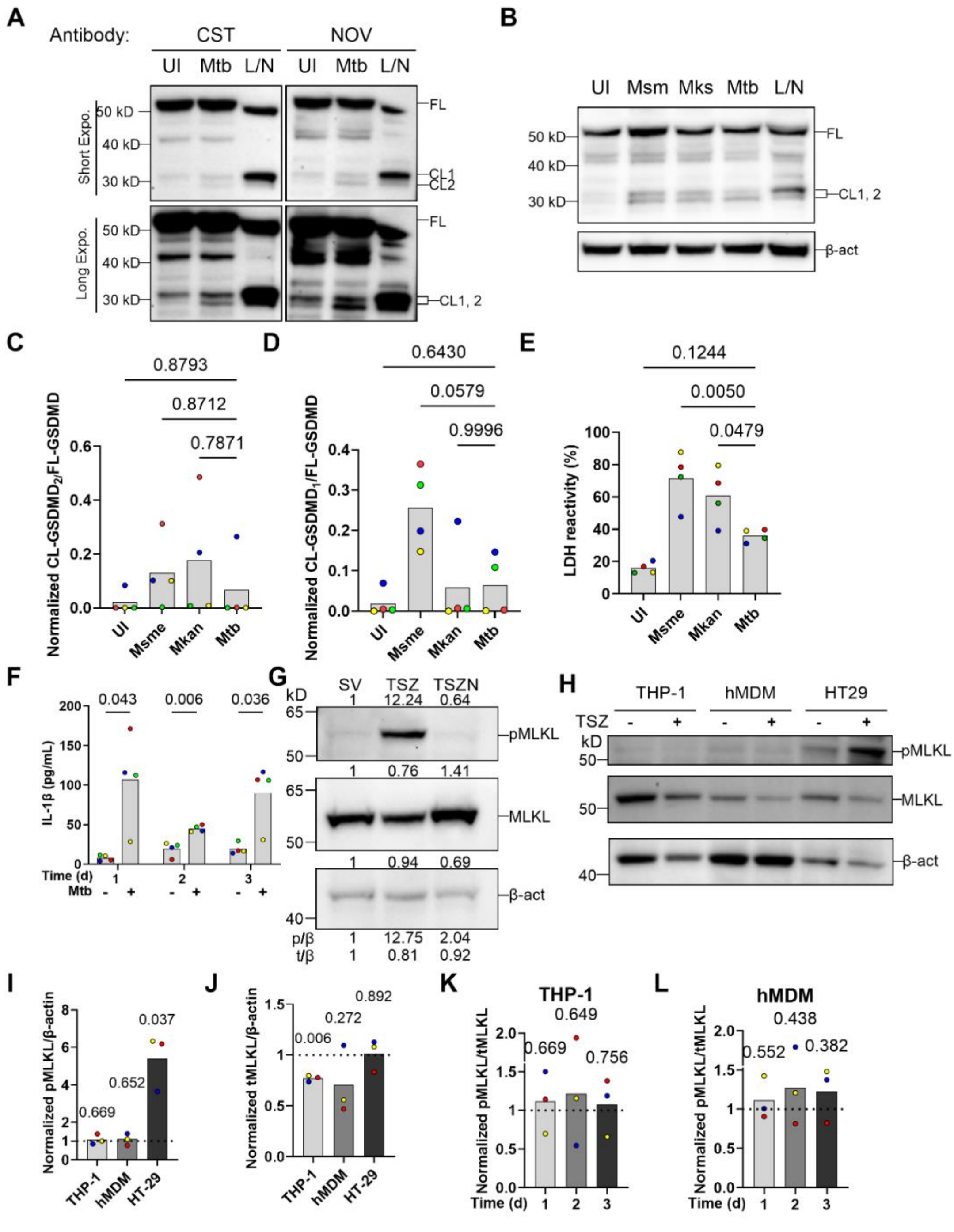

Fig. S5: Mycobacterial species induce variable GSDMD cleavage, and different cell types have distinct sensitivity to necroptosis induction. A Western blot images showing GSDMD cleavage in THP-1 cells infected with w.t. Mtb H37Rv at MOI:5 for 1 d or treated with LPS and Nigericin (L/N) or left uninfected (UI). Blots were probed using CST and NOV antibodies. Images are from one experiment (N = 1). **B** THP-1 cells were uninfected or infected with *Mycobacterium smegmatis* (Msm), *Mycobacterium kansasii* (Mkan), and Mtb, all at MOI:5 or treated with L/N. Representative western blot images showing GSDMD cleavage induced by different mycobacterial strains in THP-1 cells (N = 4). **C, D** Quantification of normalized GSDMD cleavage band intensities corresponding to

B. **E,** quantification of LDH release as a percentage of the lysis control, corresponding to **B**. Each colored data point represents an independent experiment (N = 4) for **C–E**. One-way ANOVA with Brown-Forsythe correction was performed, and *p*-values are shown in the graphs. **F** ELISA measurement of IL-1β secretion from Mtb-infected THP-1 cells (MOI:5) at 1, 2, and 3 d. Multiple unpaired t-tests with Welch's correction were performed (N = 4). **G, H** Immunoblot analysis of MLKL phosphorylation during necroptosis induction or inhibition in different cell types. SV, solvent control (0.3% DMSO); TSZ, TNF-α (100 ng/mL, O/N), SM-164 (10 μM, O/N), and zVAD-FMK (20 μM, O/N); TSZN, TSZ plus necrostatin-1 (10 μM, O/N). **G,** HT-29 cells were treated with SV, TSZ, or TSZN (N = 1). Band intensities were quantified using ImageLab software and normalized to SV for each target protein; normalized values are shown above the corresponding bands. **H** THP-1 cells, hMDMs, or HT-29 cells were treated with SV or TSZ. Quantification of normalized band intensities corresponding to phosphorylated MLKL is shown. Values from TSZ-treated samples were normalized to their SV-treated counterparts from the same experiment. Band intensities were quantified using ImageLab software. One-sample t-tests were performed to compare normalized values to 1, and *p*-values are shown in the graphs. Each colored data point represents an independent experiment using THP-1 cells or a distinct human donor for hMDMs (N = 3). **K** and **L** Quantification of normalized band intensities corresponding to phosphorylated MLKL from **Fig. 3F** or **H**, respectively. One-sample t-tests were performed to compare normalized values to 1, and *p*-values are shown in the graphs. Each colored data point represents an independent experiment (N = 3) for THP-1 cells (**Fig. 3F**) or distinct human donors (N = 3) for hMDMs (**Fig. 3H**).

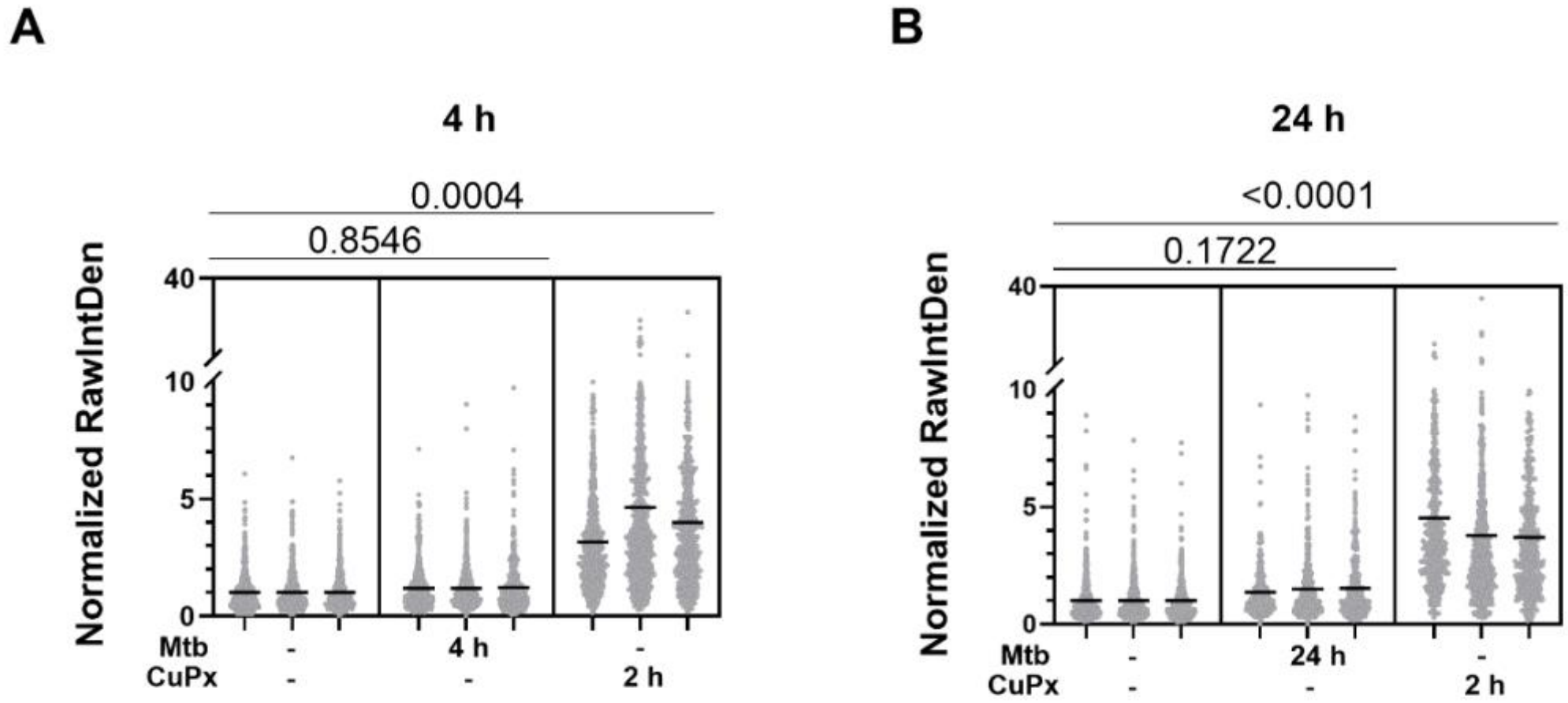

Fig. S6. Nested plot showing normalized Liperfluo fluorescence intensities in THP-1 cells. A, B Corresponding to **Fig. 4F** and **H**, Nested plots displaying Liperfluo RawIntDen quantification in individual THP-1 cells. Each dot represents a single cell, and each column corresponds to an independent experiment (N = 3). The number of cells per experiment ranges from 339 to 656. Black bars indicate the mean fluorescence intensity for each experiment. Statistical comparisons were performed using nested one-way ANOVA, and *p*-values are presented in the graphs.

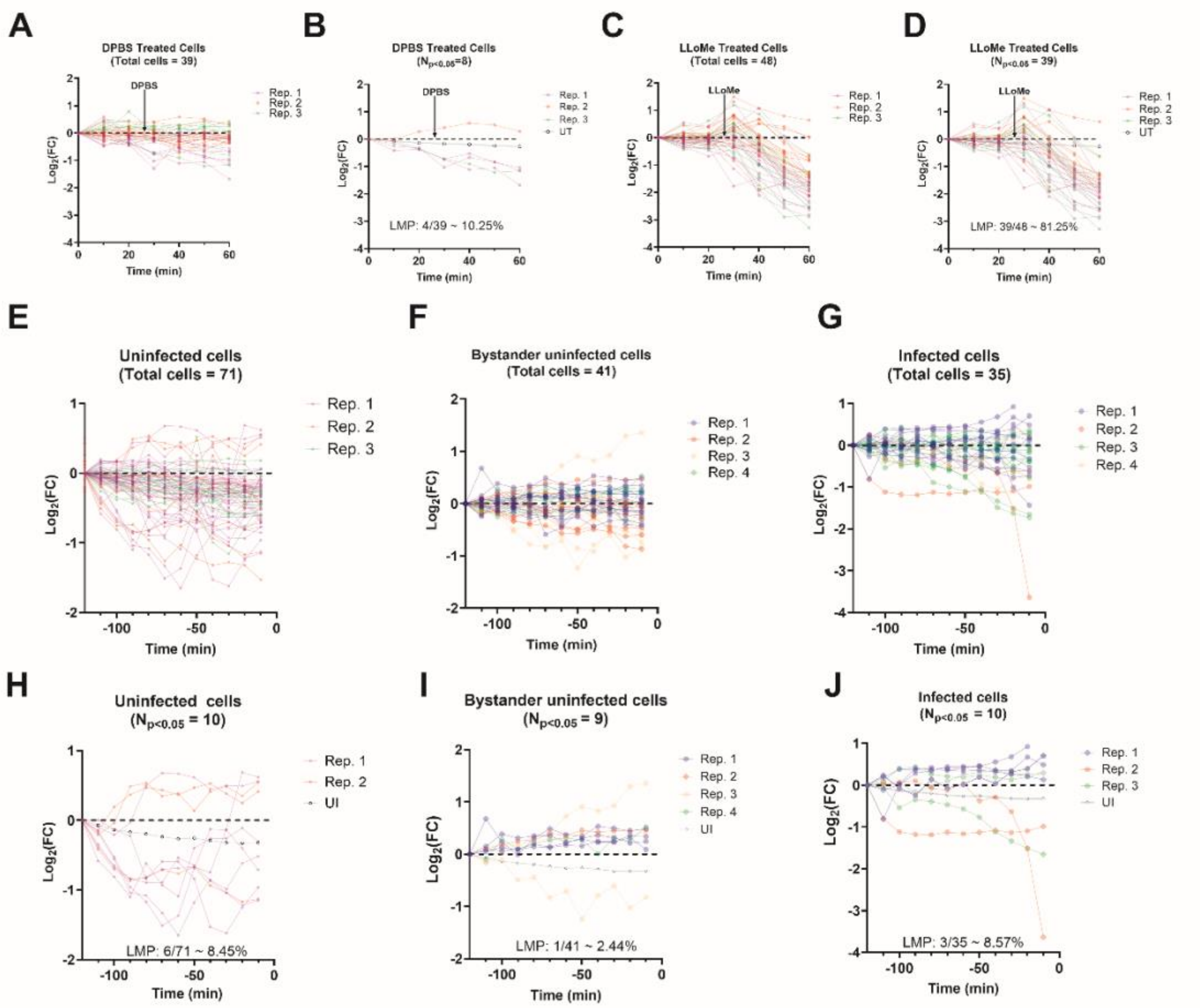

Fig. S7: Mtb does not induce detectable lysosomal membrane permeabilization (LMP) in THP-1 cells 2 hours before cell death induction. A-J The log (base 2) of normalized Lysoview (LV) fluorescence intensity was plotted against time. The grey curves represent the mean of the UI/UT condition, which served as a baseline for comparison. Each data point indicates the fluorescence value at a specific time point for a single cell with each color representing an independent experiment (N = 3). Each curve represents the time course of a single cell. The black arrow marks the time of DPBS or LLoMe addition. The number of total individual cells tracked in each experiment is shown in the title of each graph. **B, D, H, I,** and **J** are subsets of the datasets shown in **A**, **C**, **E**, **F**, and **G**, respectively, highlighting individual curves that exhibit significant differences relative to the baseline. The proportion of descending curves relative to the total number in each category was calculated and reported as the percentage of LMP.

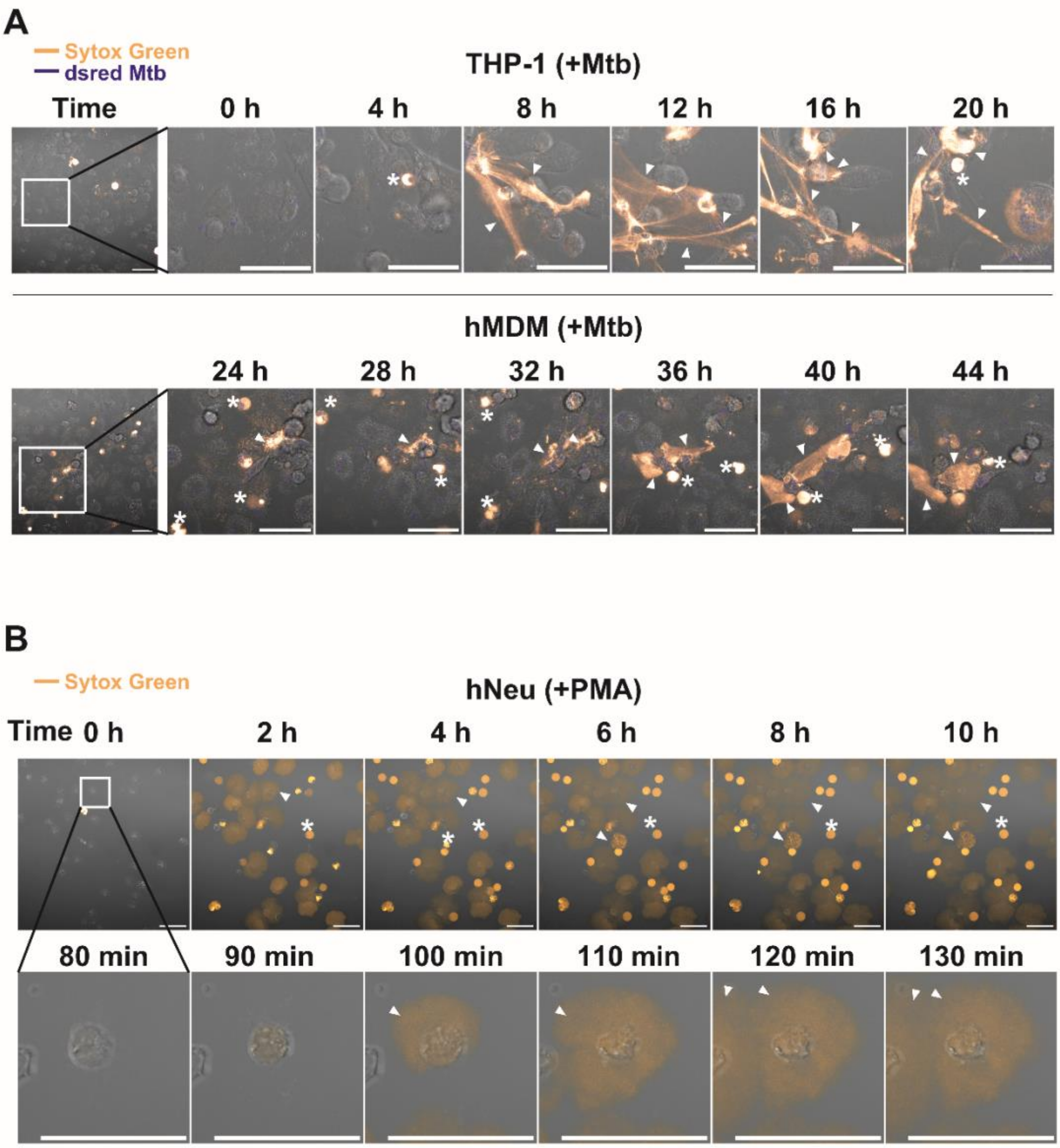

Fig. S8: Zoomed-in images illustrating the structures of released or expelled DNA. A Zoomed-in images corresponding to **Fig. 6A** showing the structures of released DNA from Mtb-infected THP-1 cells or hMDMs. **B** Time-lapse live-cell imaging of PMA-treated human neutrophils (hNeu) (N = 1). Image acquisition began immediately after the addition of 50 ng/mL PMA to hNeu. White arrowheads indicate extracellular, web-like DNA structures, while asterisks label cells that do not exhibit DNA release. Scale bars represent 50 µm.

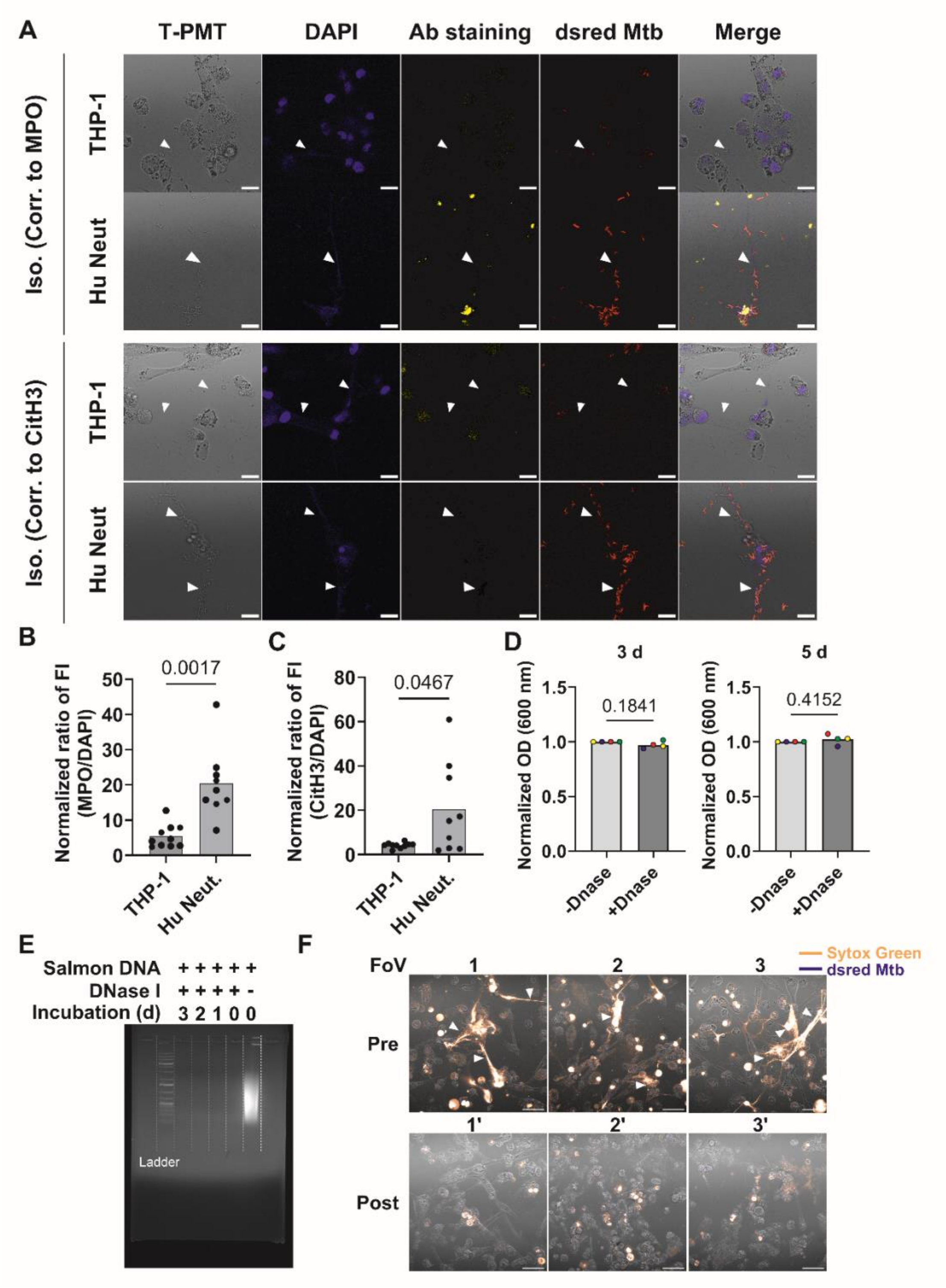

Fig. S9: Isotype controls for anti-MPO and anti-CitH3 antibody staining and validation of DNase I efficacy. A Representative confocal immunofluorescence images of dsRed H37Rv Mtb-infected THP-1 cells and hNeu undergoing DNA release. Cells were infected with dsRed H37Rv Mtb (red) at MOI: 2 for 20 hours, then fixed and stained with DAPI (blue) to visualize DNA. Alexa Fluor 647-conjugated mouse or rabbit IgG isotype control antibodies (yellow) were used to assess background staining. White arrowheads indicate extracellular web-like DNA structures. Scale bars: 20 µm. **B, C** Quantification of

the normalized fluorescence intensity (FI) ratios. Each dot represents a single extracellular DNA domain. A total of 9 or 10 domains from 3 independent experiments (N = 3) were included in the analysis. Statistical comparisons were performed using unpaired t-tests with Welch's correction. One outlier from the hNeu group in **C** was identified and removed by the ROUT test (Q = 1%). **D** Comparisons of ODs between DNase I-treated and untreated Mtb 7H9 cultures. Well-grown Mtb cultures were diluted to OD_{600} = 0.2 from different initial ODs. DNase I was added to the cultures at 100 U/mL, and the cultures were shaken at 140 rpm at 37 °C. OD_{600} values were measured at 3 and 5 d post-DNase treatment (N = 4). For each repeat, values from DNase-treated cultures were normalized to their untreated counterparts. **E** Analysis of DNase I activity in THP-1 culture medium at different time points by agarose gel electrophoresis. DNase I was diluted in RPMI 1640 medium containing 10% FBS to 100 U/mL and incubated at 37 °C for 0, 1, 2, or 3 days. After the incubation, 10 μL of the pre-incubated DNase I solutions from all groups were mixed with 10 μg (1 μL) of shredded salmon sperm DNA and incubated at 37 °C for 1 hour. Then the reaction mixtures were mixed with loading buffer and analyzed by agarose gel electrophoresis (N = 1). **F** Confocal fluorescence images of Mtb-infected THP-1 cells pre- and post-DNase I treatment. THP-1 cells were infected with dsRed H37Rv Mtb (blue) at MOI 5. 4 h after bacterial addition (defined as time 0), cells were washed twice with DPBS and replenished with fresh culture medium. At 24 h post-infection, cells were stained with Syt G (orange) and imaged, with three random fields of view (FoVs) acquired. DNase I (100 U/mL) was then added to the same well, and cells were incubated for 1 h at 37 °C under 5% CO_2. Three additional FoVs from the same well were imaged post-DNase treatment using identical microscope settings (N = 1).

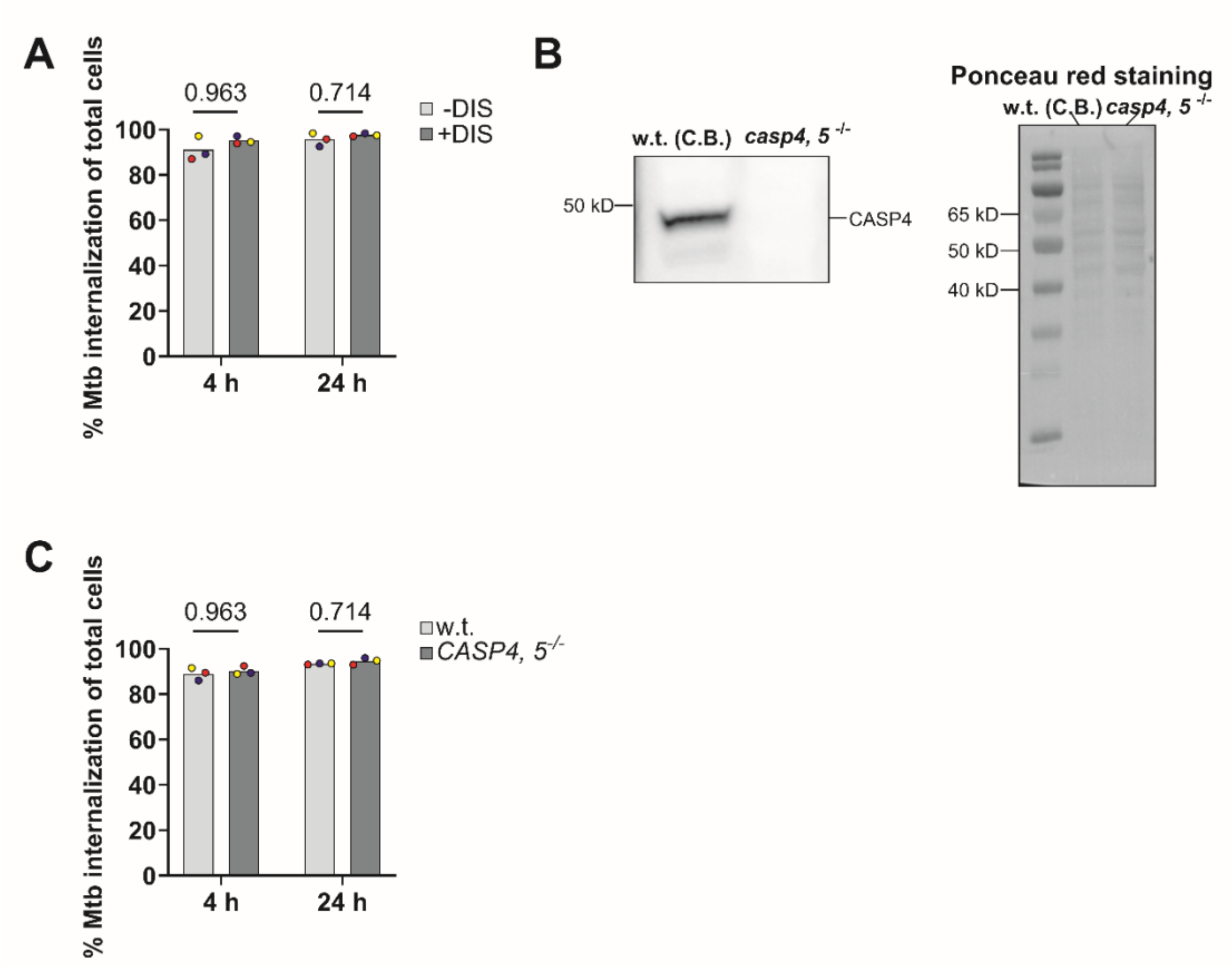

Fig. S10: Comparison of phagocytic capacity among different cell groups and validation of *CASP4/5* genotype by immunoblotting. A, C Quantification of the percentage of Mtb internalization relative to the total cells in corresponding frames (N = 3). Welch's t-tests were performed, and *p*-values are shown in the graph. **B** Immunoblot and Ponceau red staining confirming the genotype of THP-1 cell lines (N = 1). THP-1 cells were differentiated and left untreated.

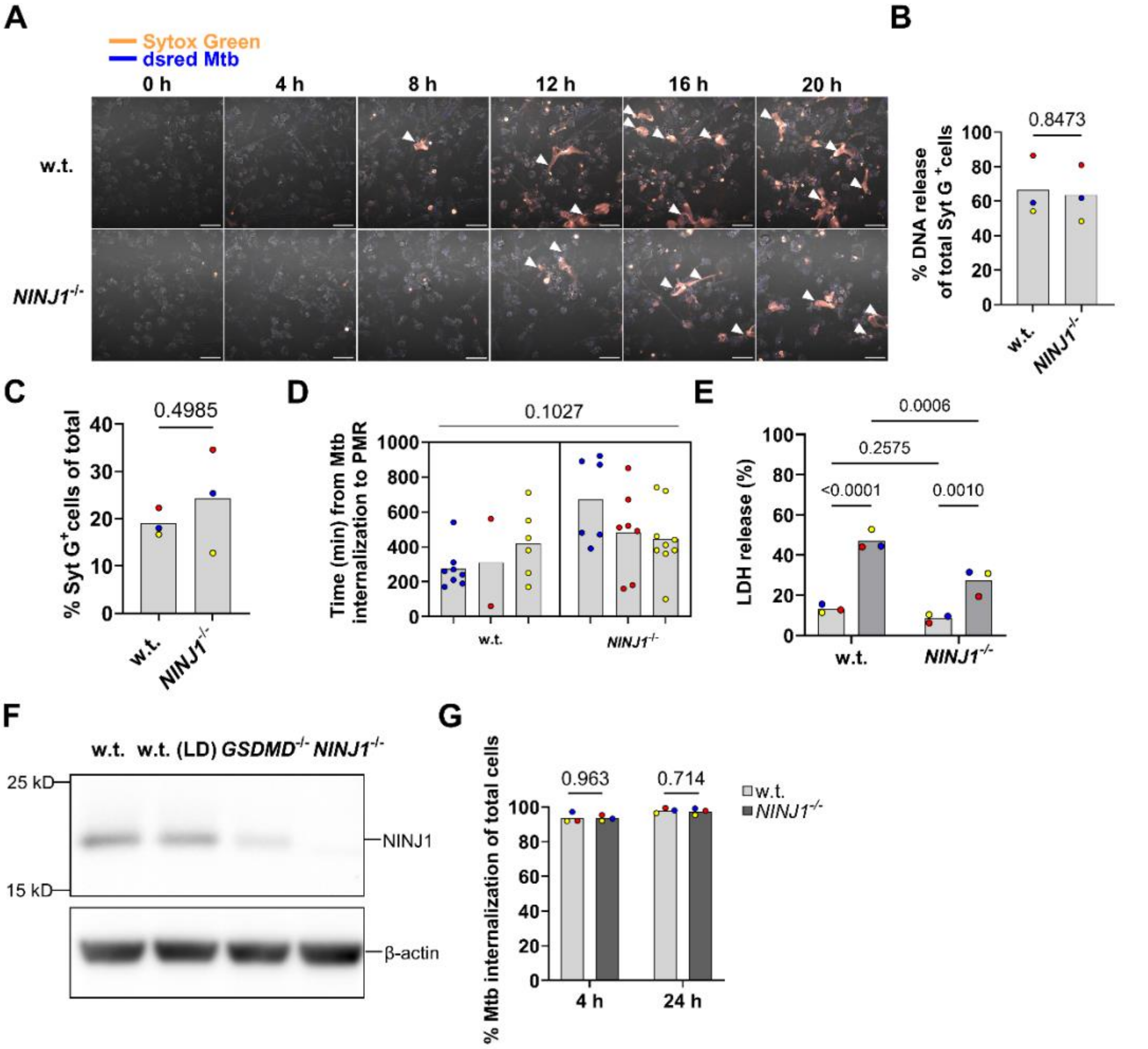

Fig. S11: NINJ1 promotes cell lysis of Mtb-infected THP-1 cells but is not required for DNA release. **A** Representative confocal time-lapse images showing Mtb-infected *NINJ1*$^{-/-}$ and w.t. THP-1 cells undergoing DNA release. *NINJ1*$^{-/-}$ and its w.t. counterpart are kind gifts from Dr. Liron David. Cells were infected with dsRed H37Rv Mtb (blue) at MOI:2 for 20 hrs and stained with Syt G (orange) to visualize DNA. White arrowheads indicate extracellular web-like DNA structures. Scale bars represent 50 µm. **B, C** Quantification of the percentage of DNA release (**B**) and Syt G^{+} cells (**C**). Welch's t-tests were applied for statistical analysis. **D** Quantification of time durations from Mtb internalization to PMR. A nested t-test was performed. **E** LDH release relative to the lysis control. Two-way ANOVA was applied for statistical analysis. **F** Immunoblot confirming *NINJ1* genotype in differentiated, untreated THP-1 cells (*N* = 1). LD, initials of Liron David. **G** Quantification of the percentage of Mtb internalization relative to the total cells in corresponding frames. Welch's t-tests were performed. Experiments were repeated 3 times (N = 3), and *p*-values were reported in the graphs for **B**, **C**, **D**, **E**, and **G**.
